# Molecular identification of *Bulinus* spp. intermediate host snails of *Schistosoma* spp. in crater lakes of western Uganda with implications for the transmission of the *Schistosoma haematobium* group parasites

**DOI:** 10.1186/s13071-019-3811-2

**Published:** 2019-11-27

**Authors:** Immaculate Tumwebaze, Catharina Clewing, Marie Claire Dusabe, Julius Tumusiime, Grace Kagoro-Rugunda, Cyril Hammoud, Christian Albrecht

**Affiliations:** 1 Department of Animal Ecology and Systematics, Justus Liebig University Giessen, Giessen, Germany; 2 Rwanda Wildlife Conservation Association, Kigali, Rwanda; 3 Department of Biology, Mbarara University of Science and Technology, Mbarara, Uganda; 4 Department of Biology, Royal Museum for Central Africa, Leuvensesteenweg 13, 3080 Tervuren, Belgium; 5 Limnology Research Unit, Ghent University, K. L. Ledeganckstraat 35, 9000 Ghent, Belgium

**Keywords:** *Bulinus forskalii*, *Bulinus tropicus*, *Bulinus truncatus*, *Schistosoma haematobium*, *Schistosoma bovis*, Neglected tropical disease, Schistosomiasis surveillance

## Abstract

**Background:**

Human schistosomiasis is the second most important tropical disease and occurs in two forms in Africa (intestinal and urogenital) caused by the digenetic trematodes *Schistosoma mansoni* and *Schistosoma haematobium*, respectively. A proposed recent shift of schistosomiasis above a previously established altitudinal threshold of 1400 m above sea level in western Ugandan crater lakes has triggered more research interest there.

**Methods:**

Based on extensive field sampling in western Uganda and beyond and employing an approach using sequences of the mitochondrial barcoding gene cytochrome *c* oxidase subunit 1 (*cox*1) this study aims were: (i) identification and establishment of the phylogenetic affinities of *Bulinus* species as potential hosts for *Schistosoma* spp.; (ii) determining diversity, frequency and distribution patterns of *Bulinus* spp.; and (iii) establishing genetic variability and phylogeographical patterns using Bayesian inference and parsimony network analyses.

**Results:**

Out of the 58 crater lakes surveyed, three species of *Bulinus* snails were found in 34 crater lakes. *Bulinus tropicus* was dominating, *Bulinus forskalii* was found in two lakes and *Bulinus truncatus* in one. The latter two species are unconfirmed potential hosts for *S. haematobium* in this region. However, *Bulinus tropicus* is an important species for schistosomiasis transmission in ruminants. *Bulinus tropicus* comprised 31 haplotypes while both *B. forskalii* and *B. truncatus* exhibited only a single haplotype in the crater lakes. All species clustered with most of the haplotypes from surrounding lake systems forming source regions for the colonization of the crater lakes.

**Conclusions:**

This first detailed malacological study of the crater lakes systems in western Uganda revealed presence of *Bulinus* species that are either not known or not regionally known to be hosts for *S. haematobium*, the causing agent of human urogenital schistosomiasis. Though this disease risk is almost negligible, the observed dominance of *B. tropicus* in the crater lakes shows that there is a likelihood of a high risk of infections with *Schistosoma bovis*. Thus, extra attention should be accorded to safeguard wild and domestic ruminants in this region as the population benefits from these animals.

## Background

Schistosomiasis is an important tropical disease especially in sub-Saharan Africa, with more than 90% of the disease burden [1] and the second most important public health disease after malaria [1, 2]. Schistosomiasis is a parasitic disease transmitted by planorbid gastropods. Human schistosomiasis in Africa occurs in two forms (intestinal and urogenital), caused by the digenetic trematodes *Schistosoma mansoni* and *Schistosoma haematobium*, respectively. Urogenital schistosomiasis accounts officially for two-thirds of all cases [3], a figure that might be too optimistic as the real prevalence of the disease is potentially underestimated by a factor of three [4]. The already important direct impact of urogenital schistosomiasis is worsened by its established role in cancer epidemics and AIDS epidemics in Africa (reviewed in [5]), besides the long recognized roles in other pathologies and diseases such as haematuria and female genital schistosomiasis (reviewed in [6]). Interestingly, *S. haematobium* is the least studied of the major human schistosomes [7, 8].

Unlike in many other regions of sub-Saharan Africa, in Uganda, intestinal rather than urogenital schistosomiasis is considered a major public health problem [9]. Urogenital schistosomiasis, though present has long been assumed to be restricted to a few areas of eastern and northern Uganda [10]. Schistosomiasis studies in Uganda have so far been focused on regional intestinal schistosomiasis [11–14] around the great lake systems of Lake Victoria and Lake Albert. A negligible amount of studies have been conducted on urogenital schistosomiasis and *S. haematobium* and their planorbid host snails belonging to the genus *Bulinus*. Today there is no official declaration about any region of Uganda to be completely free of urogenital schistosomiasis. Thus, the status of urogenital schistosomiasis remains an enigma in Uganda.

**Fig. 1:**
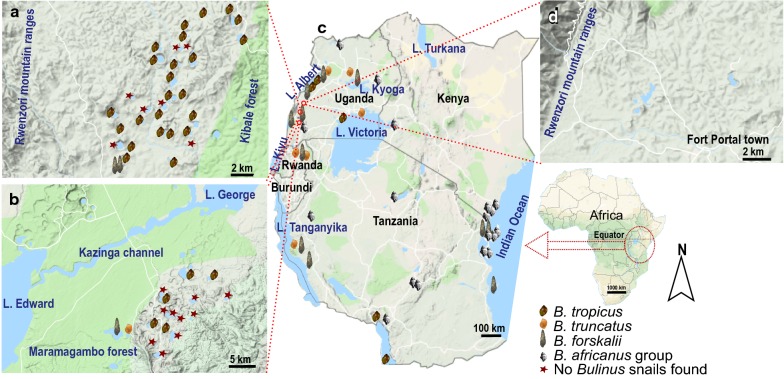
Sampling sites in the three crater lakes fields in Uganda and at the supra-regional scale. **a** Ndali-Kasenda crater lakes. **b** Bunyaruguru crater lakes. **c** Populations from East Africa. **d** Fort Portal crater lakes. Snail symbols indicate localities of *Bulinus* populations studied, whereas a star indicates when sampling did not yield a *Bulinus* population in the respective crater lake. Note that data for some populations used were retrieved from GenBank (see Table 1)

A recent study [15] has indicated that intestinal schistosomiasis actually occurs above an earlier designated threshold of 1400 m above sea level (a.s.l.), specifically in crater lakes in western Uganda. For instance, a high rate of intestinal schistosomiasis in travelers after a brief exposure to the high-altitude crater Lake Nyinambuga was reported in 2012 [16]. The Albertine Rift valley region of western Uganda is dominated by mainly two types of freshwater bodies; the three great lakes Albert, Edward, George and about 90 small crater lakes of varying sizes scattered throughout the region. The crater lakes have originally been divided into four geographical fields of Fort Portal, Ndali-Kasenda, Katwe-Kikorongo and Bunyaruguru [17] (see Fig. 1). They straddle the equator between and are spread along the regional rift valley gradient from 914 m to 1566 m elevation [18], with varying limnological characteristics [19] and climatic gradient. The region is one of the most densely populated rural areas in sub-Saharan Africa [20] and is also a tourist destination, attracting local and international travelers.

The potential of urogenital schistosomiasis in Uganda has been neglected, despite the fact that the disease is common in regions nearby such as the Democratic Republic of the Congo (DRC) [21], Tanzania [22] and South Sudan [23]. It has recently been shown that targeting the schistosome intermediate hosts is the most effective of all elimination strategies combating the burden of schistosomiasis [24]. A first step in targeting regional transmission foci is the correct identification of the intermediate hosts [25]. This is particularly true for the *Bulinus* spp./*Schistosoma haematobium* system, since *Bulinus* is a very diverse freshwater gastropod genus of currently recognized 37 species belonging to four species complexes that are morphologically variable [26, 27]. Although there are still some taxonomic issues involved, it has repeatedly been shown that these species complexes can be identified using molecular genetic tools [28–31]. Three of the species complexes are known to occur in regions along the Albertine Rift, namely the *B. truncatus/B. tropicus* complex, the *B. forskalii* group and the *B. africanus* group [32]. These regions are potentially species source pools for the crater lakes. Given that these groups include potential hosts for *S. haematobium*, it is important to survey the crater lakes region for snails transmitting human urogenital schistosomiasis. Therefore, enhanced simultaneous mapping and monitoring of strains of urogenital schistosomiasis and their intermediate hosts populations are both necessary to control this disease in areas where it has not been known to occur before. Very little information, however, exists on the mollusc fauna of the crater lakes [33]. This necessitates an assessment of the status of potential host snail species and thus urogenital schistosomiasis in the region.

Based on extensive field sampling in the crater field region and beyond and employing an approach using sequences of the barcoding gene of mitochondrial cytochrome *c* oxidase subunit 1 (*cox*1) this study aims are: (i) identification and establishment of the phylogenetic affinities of *Bulinus* species as potential hosts for *Schistosoma* spp.; (ii) determining diversity, frequency and distribution patterns of *Bulinus* spp.; and (iii) establishing genetic variability and phylogeographical patterns.

## Methods

### Study area

**Fig. 2:**
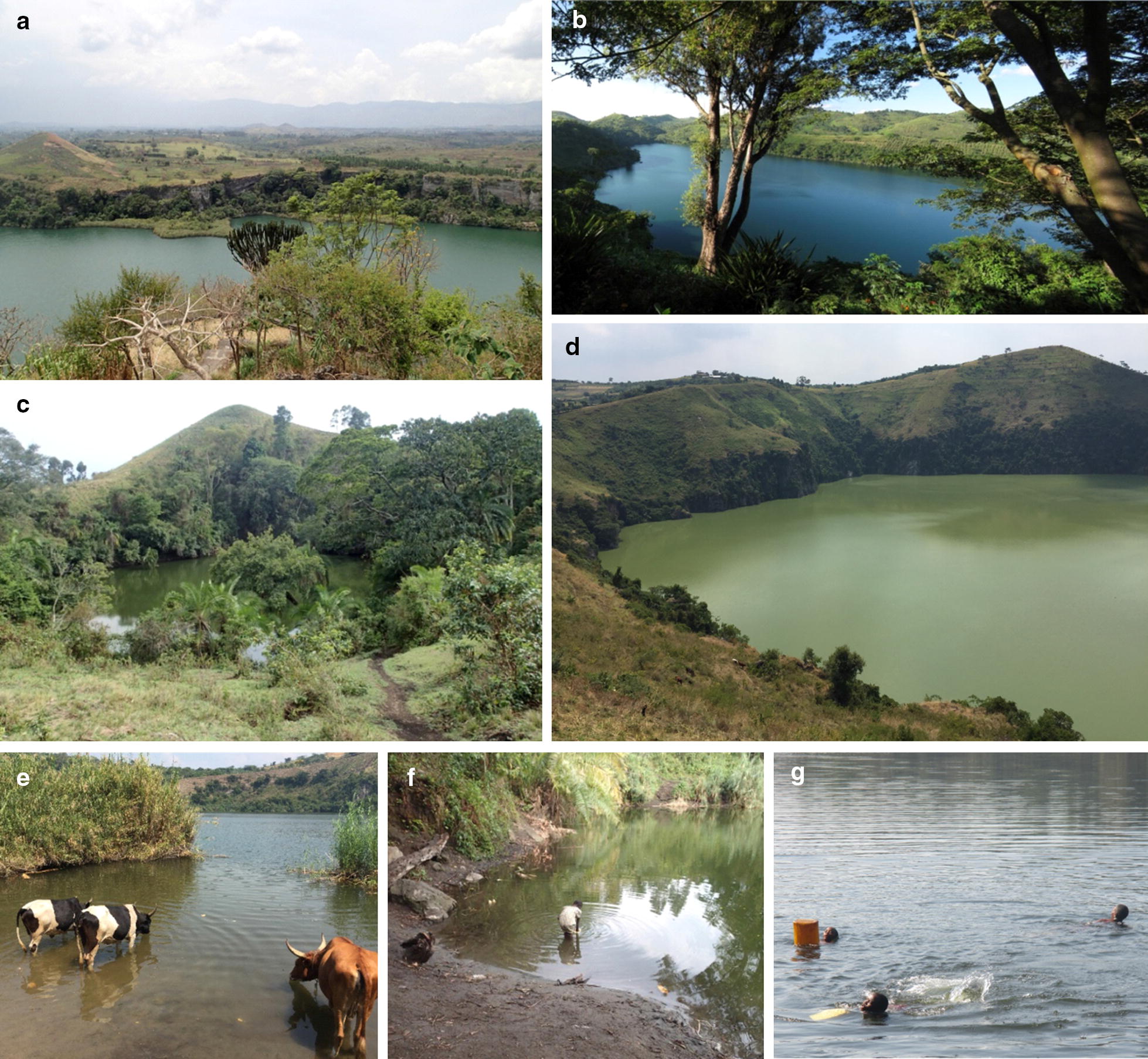
Impressions from selected crater lakes in western Uganda. **a** Lake Kyaninga (Fort Portal). **b** Lake Nyinambuga (Ndali-Kasenda). **c** Lake Ekikoto (Fort Portal). **d** Lake Ntambi (Ndali-Kasenda). **e** Lake Nyamugosani (Ndali-Kasenda). **f** Lake Kayihara (Fort Portal). **g** Lake Kako (Bunyaruguru) (Photo credit: C. Albrecht (**a**, **c**, **f**); D. Engelhardt (**b**); C. Dusabe (**d**, **e**); I. Tumwebaze (**g**))

This study was conducted in lakes of the three main crater fields in Uganda, between Fort Portal region in the north, Ndali-Kasenda in the middle and Bunyaruguru in the south (Fig. 1). The region is bordered by the vast Rwenzori Mountains in the north-west, the southern shores of Lake Albert in the north and the region of the Queen-Elizabeth National Park (Lake Edward-George) in the south. Most of the crater lakes were formed by faulting and volcanic eruption some 8000 to 10,000 years ago [34]. Bunyaruguru lakes lie on the southern side of the Edward-George system while the rest are located on its northern side. The climate, hydrology, water chemistry and landscape settings are highly heterogeneous between crater fields. Lakes in Fort Portal crater field lie at higher altitude (above 1500 m a.s.l), than those in the Ndali-Kasenda crater field and Bunyaruguru. Lake Kyaninga (Fig. 2a) is one of the deepest (220 m) known crater lake in western Uganda [35], although the crater lakes are generally shallow. Some of the lakes are embedded in a still rather natural setting whereas the surroundings of many of the lakes studied are highly disturbed by anthropogenic activities. The lakes are commonly exploited as a water source for humans and livestock consumption (Fig. 2). Furthermore, these lakes are also a main source of food for the local communities using them for fishing. Some of the fish species are introduced. For example, *Tilapia zillii*, *Orechromis leucosticus* and *Poecilia reticulata* were introduced in Lake Nkuruba [36].

### Sampling

Out of 58 crater lakes sampled, 19 were from Bunyaruguru, 33 from Ndali-Kasenda and the 6 remaining from Fort Portal crater field (Table 1). We purposively selected these lakes to cover a range from low altitude (1033 m a.s.l) to high altitude (1569 m a.s.l). The selection of a lake and/or sampling site was based on representation of lake field size, lake size class, utilization and lake type as well as accessibility. Lakes of Katwe-Kikorongo field are known to be mainly saline [19] and were therefore not included in this study.

**Table 1:** Summary on localities and haplotypes found in the crater lakes and other regions studied

Crater field	Location	Lake code	Coordinates		Altitude (m)	Species	No. of haplotypes^a^	Specimen code^a^	Specimen voucher	Prep. no.	GenBank ID [reference]
			Latitude	Longitude							
Bunyaruguru	Lake Bugwagyi, Uganda	BGJ	−0.19680°N	30.18663°E	1070	*B. tropicus*	1	BGJ1.1	UGSB 21302	25644	MN551518
						*B. tropicus*		BGJ1.2	UGSB 21303	25645	MN551519
	Lake Chema^b^, Uganda		−0.25229°N	30.11444°E	1268	–			–	–	–
	Lake Kabarogyi^b^, Uganda		−0.22692°N	30.21375°E	1352	–			–	–	–
	Lake Kako^b^, Uganda		−0.30484°N	30.09728°E	1405	–			–	–	–
	Lake Kamunzuku^b^, Uganda		−0.26422°N	30.15410°E	1272	–			–	–	–
	Lake Kamweru^b^, Uganda		−0.26023°N	30.12223°E	1270	–			–	–	–
	Lake Kariya^b^, Uganda		−0.23010°N	30.16624°E	1256	–			–	–	–
	Lake Kasiriya^b^, Uganda		−0.25673°N	30.13180°E	1265	–			–	–	–
	Lake Katinda^b^, Uganda		−0.22237°N	30.10978°E	1033	–			–	–	–
	Lake Kigezi, Uganda	KGZ	−0.28589°N	30.11084°E	1323	*B. tropicus*	1	KGZ1.1	UGSB 21308	25648	MN551520
						*B. tropicus*		KGZ1.2	UGSB 21309	25649	MN551521
	Lake Kyamwiga, Uganda	KMG	−0.19196°N	30.15009°E	1035	*B. tropicus*	1	KMG1.1	UGSB 21296	25640	MN551516
						*B. tropicus*		KMG1.2	UGSB 21297	25641	MN551517
	Lake Kyasanduka, Uganda		−0.28745°N	30.04731°E	1016	*B. truncatus*			UGSB 23628	27178	MN551575
						*B. truncatus*			UGSB 23629	27179	MN551576
	Lake Mafuro, Uganda	MFR	−0.26749°N	30.10385°E	1279	*B. tropicus*	3	MFR1.1	UGSB 21287	25634	MN551510
						*B. tropicus*		MFR1.2	UGSB 21288	25635	MN551511
						*B. tropicus*		MFR2.1			HQ121571 [32]
	Lake Mugogo^b^, Uganda		−0.28262°N	30.12591°E	1323					–	
	Lake Murambi, Uganda	MRM	−0.22512°N	30.10789°E	1079	*B. tropicus*		MRM1.1	UGSB 21290	25636	MN551512
						*B. tropicus*		MRM1.2	UGSB 21291	25637	MN551513
	Lake Nyamusingire^b^, Uganda		−0.28455°N	30.03994°E	985	–			–	–	–
	Lake Nkugute^b^, Uganda		−0.32068°N	30.10341°E	1404	–			–	–	–
	Lake Nyungu, Uganda	NGU	−0.25500°N	30.09524°E	1196	*B. tropicus*	2	NGU1.1	UGSB 21293	25638	MN551513
						*B. tropicus*		NGU1.2	UGSB 21294	25639	MN551515
						*B. tropicus*		NGU2.1			HQ121571 [32]
	Lake Rwijongo^c^, Uganda	RWG	−0.27122°N	30.08909°E	1262	*B. tropicus*	2	RWG2.2			HQ121570 [32]
						*B. tropicus*		RWG2.3			HQ121571 [32]
Ndali Kasenda	Lake Kanyabuterere^b^, Uganda		0.41485°N	30.28823°E	1201	–			–	–	–
	Lake Kanyamukali, Uganda	KML	0.40699°N	30.23669°E	1167	*B. tropicus*	2	KML1.1	UGSB 21434	25839	MN551526
						*B. tropicus*		KML1.2	UGSB 21435	25840	MN551527
						*B. tropicus*		KML1.3	UGSB 16767	22523	MN551503
						*B. tropicus*		KML1.4	UGSB 16768	22524	MN551504
						*B. tropicus*		KML1.5	UGSB 16853	22571	MN551508
	Lake Kanyangwe, Uganda	KAG	0.45041°N	30.27586°E	1280	*B. tropicus*	3	KAG1.1	UGSB 22111	26335	MN551554
						*B. tropicus*		KAG1.2	UGSB 22112	26336	MN551555
						*B. tropicus*		KAG2.1			HQ121576 [32]
	Lake Kasenda, Uganda	KSD	0.43285°N	30.29179°E	1248	*B. tropicus*	5	KSD1.1	UGSB 21431	25837	MN551524
						*B. tropicus*		KSD1.2	UGSB 21432	25838	MN551525
						*B. tropicus*		KSD2.1			HQ121577 [32]
						*B. tropicus*		KSD2.2			HQ121578 [32]
						*B. tropicus*		KSD2.3			HQ121579 [32]
	Lake Kibungo, Uganda	KIG	0.39237°N	30.23338°E	1140	*B. forskalii*	1	KIG1.1	UGSB 22375	26620	MN551573
	Lake Kifuruka^b^, Uganda		0.48912°N	30.28845°E	1407	–			–	–	–
	Lake Kitere, Uganda	KTR	0.39731°N	30.27346°E	1144	*B. tropicus*	2	KTR1.1	UGSB 21570	25956	MN551541
						*B. tropicus*		KTR1.2	UGSB 21571	25957	MN551542
	Lake Kyanga, Uganda	KYG	0.40022°N	30.23221°E	1167	*B. tropicus*	2	KYG1.1	UGSB 21455	25853	MN551532
						*B. tropicus*		KYG1.2	UGSB 21456	25854	MN551533
	Lake Lugembe, Uganda	LGB	0.44722°N	30.28123°E	1298	*B. tropicus*	1	LGB1.1	UGSB 22123	26343	MN551560
						*B. tropicus*		LGB1.2	UGSB 22124	26344	MN551561
	Lake Lyantonde^b^, Uganda		0.48662°N	30.28024°E	1404	–			–	–	–
	Lake Mirambi, Uganda	MRB	0.38902°N	30.22906°E	1144	*B. forskalii*	1	MRB1.1	UGSB 22120	26341	MN551559
						*B. forskalii*		MRB1.2	UGSB 22372	26618	MN551574
	Lake Mubiro^b^, Uganda		0.44144°N	30.25545°E	1212	–			–	–	–
	Lake Muligamire, Uganda	MRR	0.42302°N	30.28884°E	1208	*B. tropicus*	1	MRR1.1	UGSB 21561	25950	MN551538
						*B. tropicus*		MRR1.2	UGSB 21562	25951	MN551539
	Lake Mwamba, Uganda	MBA	0.45746°N	30.27303°E	1307	*B. tropicus*	4	MBA1.1	UGSB 22093	26323	MN551547
						*B. tropicus*		MBA1.2	UGSB 22364	26610	MN551571
						*B. tropicus*		MBA1.3	UGSB 22365	26611	MN551570
						*B. tropicus*		MBA2.1			HQ121575 [32]
	Lake Mwengenyi, Uganda	MGY	0.48757°N	30.25972°E	1410	*B. tropicus*	2	MGY1.1	UGSB 22117	26339	MN551557
						*B. tropicus*		MGY1.2	UGSB 22118	26340	MN551558
	Lake Ndicho^b^, Uganda		0.44525°N	30.26904°E	1272	–			–	–	–
	Lake Njarayabana, Uganda	NJN	0.42805°N	30.24747°E	1204	*B. tropicus*	2	NJN1.1	UGSB 22366	26612	MN551569
						*B. tropicus*		NJN1.2	UGSB 22367	26613	MN551568
	Lake Nkuruba, Uganda	NRB	0.51720°N	30.30324°E	1517	*B. tropicus*	6	NRB1.1	UGSB 21549	25942	MN551534
						*B. tropicus*		NRB1.2	UGSB 21550	25943	MN551535
						*B. tropicus*		NRB1.3	UGSB 16761	22517	MN551501
						*B. tropicus*		NRB1.4	UGSB 16762	22518	MN551502
						*B. tropicus*		NRB2.1			HQ121568 [32]
						*B. tropicus*		NRB2.2			HQ121567 [32]
						*B. tropicus*		NRB2.3			HQ121566 [32]
						*B. tropicus*		NRB2.4			HQ121569 [32]
	Lake Ntambi^b^, Uganda		0.40729°N	30.22946°E	1155	–			–	–	–
	Lake Ntanda/Katanda, Uganda	KTD	0.47775°N	30.26165°E	1341	*B. tropicus*	1	KTD1.1	UGSB 22108	26333	MN551552
						*B. tropicus*		KTD1.2	UGSB 22109	26334	MN551553
	Lake Nyabikere, Uganda	NKR	0.50101°N	30.32792°E	1389	*B. tropicus*	3	NKR1.1	UGSB 21440	25843	MN551528
						*B. tropicus*		NKR1.2	UGSB 21441	25844	MN551529
						*B. tropicus*		NKR2.1			HQ121572 [32]
						*B. tropicus*		NKR2.2			HQ121574 [32]
	Lake Nyahira, Uganda	NHR	0.49914°N	30.28737°E	1458	*B. tropicus*	1	NHR1.1	UGSB 21552	25944	MN551536
						*B. tropicus*		NHR1.2	UGSB 21553	25945	MN551537
	Lake Nyamirima, Uganda	NMM	0.52001°N	30.32035°E	1497	*B. tropicus*	2	NMM1.1	UGSB 21720	26080	MN551543
						*B. tropicus*		NMM1.2	UGSB 21721	26081	MN551544
						*B. tropicus*		NMM2.1			HQ121568 [32]
	Lake Nyamugosani, Uganda	NGS	0.42498°N	30.23139°E	1237	*B. tropicus*	2	NGS1.1	UGSB 21428	25835	MN551522
						*B. tropicus*		NGS1.2	UGSB 21429	25836	MN551523
						*B. tropicus*		NGS1.3	UGSB 22099	26327	MN551550
	Lake Nyamugoro^b^, Uganda		0.44809°N	30.24233°E	1272	–			–	–	–
	Lake Nyamuteza, Uganda	NTZ	0.43509°N	30.22866°E	1261	*B. tropicus*	2	NTZ1.1	UGSB 21734	26089	MN551545
						*B. tropicus*		NTZ1.2	UGSB 21735	26090	MN551546
	Lake Nyanswiga, Uganda	NSG	0.50733°N	30.28825°E	1479	*B. tropicus*	1	NSG1.1	UGSB 21567	25954	MN551540
						*B. tropicus*		NSG1.2	UGSB 22359	26606	MN551572
	Lake Nyinabulita, Uganda	NBT	0.50760°N	30.32533°E	1424	*B. tropicus*	2	NBT1.1	UGSB 21446	25847	MN551530
						*B. tropicus*		NBT1.2	UGSB 21447	25848	MN551531
	Lake Nyinambuga, Uganda	NNG	0.48109°N	30.28773°E	1372	*B. tropicus*	3	NNG1.1	UGSB 22106	26332	MN551551
						*B. tropicus*		NNG1.2	UGSB 22368	26614	MN551567
						*B. tropicus*		NNG1.3	UGSB 22369	26615	MN551566
						*B. tropicus*		NNG1.4	UGSB 17784	23160	MN551500
						*B. tropicus*		NNG2.1			HQ121573 [32]
	Lake Rukwanzi, Uganda	RKZ	0.47481°N	30.27925°E	1345	*B. tropicus*	1	RKZ1.1	UGSB 22115	26338	MN551556
						*B. tropicus*		RKZ1.2	UGSB 22370	26616	MN551565
						*B. tropicus*		RKZ1.3	UGSB 22371	26617	MN551564
	Lake Rwandakara, Uganda	RKR	0.41567°N	30.27054°E	1171	*B. tropicus*	2	RKR1.1	UGSB 22126	26345	MN551562
						*B. tropicus*		RKR1.2	UGSB 22127	26346	MN551563
	Lake Rwenjuba, Uganda	RJB	0.43922°N	30.26577°E	1257	*B. tropicus*	2	RJB1.1	UGSB 22096	26325	MN551548
						*B. tropicus*		RJB1.2	UGSB 22097	26326	MN551549
	Lake Wankenzi, Uganda	WKZ	0.41834°N	30.26326°E	1158	*B. tropicus*	3	WKZ1.1	UGSB 16770	22526	MN551505
						*B. tropicus*		WKZ1.2	UGSB 16839	22557	MN551507
						*B. tropicus*		WKZ1.3	UGSB 16854	22572	MN551509
Fort Portal	Lake Balama^b^, Uganda		0.67959°N	30.23345°E	1566				–	–	–
	Lake Ekikooto^b^, Uganda		0.70142°N	30.31342°E	1536				–	–	–
	Lake Kayihara^b^, Uganda		0.70256°N	30.31590°E	1549				–	–	–
	Lake Kyaninga, Uganda		0.70070°N	30.29919°E	1553				–	–	–
	Lake Saaka^b^, Uganda		0.68700°N	30.23954°E	1569				–	–	–
	Lake Wabikere^b^, Uganda		0.68826°N	30.23518°E	1550				–	–	–
Others (non crater lakes)	Angola, Bengo, Cabungo					*B. globosus*					LT671918 [81]
						*B. globosus*					LT671947 [81]
	Angola, Bengo, Burgalheira escola					*B. globosus*					LT671925 [81]
	Angola, Luanda, Candimba/Muxima					*B. globosus*					LT671928 [81]
	Angola, Kwanza Norte, Cacuso					*B. globosus*					LT671936 [81]
	Angola, Luanda, Cassefo					*B. forskalii*					LT671955 [81]
	Angola, Luanda, Caquila					*B. forskalii*					LT671956 [81]
	Angola, Bengo, Porto mangueiras					*B. forskalii*					LT671950 [81]
						*B. forskalii*					LT671951 [81]
	Angola, Kwanza Norte, N’Dalatando					*B. forskalii*					LT671957 [81]
	Angola, Bengo, Icau Wando					*B. forskalii*					LT671966 [81]
						*B. forskalii*					LT671949 [81]
	Angola, Malanje, Carlanga					*B. forskalii*					LT671961 [81]
						*B. forskalii*					LT671962 [81]
	Angola, Malanje, Calandula					*B. forskalii*					LT671964 [81]
	Angola, Quifangondo					*B. forskalii*					AM286306 [30]
	Cameroon, Peptonoum-west	CAM	5.63306°N	10.63528°E		*B. tropicus*	2	CAM1.1			KJ157495 [54]
						*B. tropicus*		CAM1.2			KJ157496 [54]
						*B. tropicus*					KJ157497 [54]
	Burkina Faso, Mogtedo barrage		12.30647°N	−0.82783°E		*B. forskalii*					AM286310 [30]
						*B. globosus*					AM286293 [30]
						*B. truncatus*					AM286315 [30]
	Cameroon, Peptonoum-east					*B. tropicus*					KJ157492 [54]
						*B. tropicus*					KJ157494 [54]
	Cameroon, Bertoua		4.58889°N	13.68111°E		*B. truncatus*					KJ135287 [54]
	Cameroon, Mourtous					*B. globosus*					KJ157471 [54]
	Cameroon, Yagoua					*B. globosus*					KJ157472 [54]
	Cameroon, Kaprissi					*B. globosus*					KJ157475 [54]
	Cameroon, Gounougou					*B. globosus*					KJ157473 [54]
	Cameroon, Ouroudoukoudje					*B. globosus*					KJ157474 [54]
	Cameroon, Kaele					*B. senegalensis*					KJ157481 [54]
						*B. senegalensis*					KJ157480 [54]
	Cameroon, Kekem					*B. truncatus*					KJ135289 [54]
	Cameroon, Mokolo					*B. truncatus*					KJ135291 [54]
	Cameroon, Loum					*B. truncatus*					KJ135295 [54]
	DR Congo, Lake Kivu					*B. truncatus*					HQ121561 [32]
	Egypt, Quena		26.17306°N	32.16611°E		*B. truncatus*					KJ135304 [54]
	Giza, Egypt		30.14139°N	31.07694°E		*B. truncatus*					KJ135300 [54]
	Iran, Khouzestan					*B. truncatus*					KT365867
	Kenya, Nimbodze					*B. nasutus nasutus*					AM921841 [30]
	Kenya, Kisumu, Kandaria dam					*B. globosus*					AM286286 [30]
	Kenya, Kinango					*B. globosus*					AM921844 [30]
	Kenya, Kachetu					*B. globosus*					AM921847 [30]
	Kenya, Mwamduli					*B. globosus*					AM921850 [30]
	Malawi, Lake Malawi	LM				*B. nyassanus*	1	LM1.1			AM921838 [30]
						*B. africanus*					AM286296 [30]
	Niger, Satoni					*B. forskalii*					AM286308 [30]
						*B. truncatus*					AM286316 [30]
	Niger, Tonida					*B. globosus*					AM286294 [30]
						*B. globosus*					AM921808 [30]
	Nigeria, Ibaro, Ogun		7.15000°N	3.11667°E		*B. truncatus*					FN546781 [80]
	Nigeria, Ilie		8.25000°N	4.96667°E		*B. truncatus*					FN546797 [80]
						*B. truncatus*					FN546797 [80]
	Nigeria, Oshogbo		8.08333°N	4.66667°E		*B. truncatus*					FN546805 [80]
						*B. truncatus*					FN546805 [80]
	Nigeria, Oju Alaro					*B. globosus*					FN546815 [80]
	Nigeria, Ipogun					*B. globosus*					FN546814 [80]
	Nigeria, Imala, Odo					*B. truncatus*					FN546787 [80]
	Nigeria, Awuru					*B. truncatus*					FN546786 [80]
	Oman					*B. wrighti*					AM286318 [30]
	Rwanda, rice scheme dam lake		−1.28652°N	30.31509°E	1349	*Bulinus* sp.			UGSB 16778	22532	MN551579
						*B. truncatus*			UGSB 16777	22531	MN551578
	Rwanda, Lake Muhazi		−1.85912°N	30.49039°E	1452	*B. truncatus*			UGSB 16755	22511	MN551581
	Rwanda, Lake Muhazi		−1.84843°N	30.47826°E	1455	*B. truncatus*			UGSB 4936	22549	MN551580
	Sardinia, Posada		40.63487°N	9.67537°E		*B. truncatus*					AM286312 [30]
	Sao Tome and Principe, Sao Tome Island					*B. forskalii*					AM286305 [30]
	Senegal, Dakar					*B. forskalii*					AM286307 [30]
	Senegal, Diama		15.43611°N	16.23389°E		*B. truncatus*					KJ135306 [54]
	Senegal, Toukar					*B. senegalensis*					KJ157483 [54]
	Senegal, Diohine					*B. senegalensis*					KJ157484 [54]
						*B. senegalensis*					KJ157485 [54]
	Senegal, Poudaye					*B. senegalensis*					KJ157486 [54]
	South Africa, Lake Sibaya	SA				*B. natalensis*	1	SA1.1			AM286311 [30]
	South Africa, Pietermaritzburg					*B. globosus*					AM286289 [30]
						*B. globosus*					AM286290 [30]
	South Africa, Durban Isipingo					*B. africanus*					AM286295 [30]
	Tanzania, Muyuni, Unguja		−6.37845°N	39.46415°E		*B. nasutus productus*					AM286299 [30]
	Tanzania, Njombe Kibena	TZ	−9.20382°N	34.78402°E		*B. tropicus*	1	TZ1.1			AM921834 [30]
						*B. tropicus*		TZ1.2			AM921837 [30]
						*B. tropicus*		TZ1.3			AM921842 [30]
	Tanzania, Lake Sagara,		−5.25140°N	31.08518°E		*Bulinus* sp.					AM286298 [30]
	Tanzania, Ihayabuyaga					*B. nasutus productus*					AM286300 [30]
						*B. nasutus productus*					AM286301 [30]
	Tanzania, Njombe Rujewa					*B. nasutus productus*					AM921833 [30]
	Tanzania, Zanzibar Vitonguji, Pemba Island		−5.23378°N	39.82843°E		*B. nasutus productus*					AM921809 [30]
	Tanzania, Zanzibar, Pemba Island		−5.17120°N	39.82198°E		*B. nasutus productus*					AM921811 [30]
	Tanzania, Zanzibar, Pemba Island		−4.92803°N	39.73785°E		*Bulinus* sp.					AM921832 [30]
	Tanzania, Zanzibar, Pemba Island					*B. globosus*					MH014041 [82]
	Tanzania, Zanzibar, Pemba Island, Kangagani					*B. barthi*					AM921818 [30]
	Tanzania, Zanzibar, Mafia Island					*B. nasutus nasutus*					AM921831 [30]
	Tanzania, Zanzibar, Mafia Island, Kanga swamp					*B. barthi*					AM921814 [30]
						*B. globosus*					AM921823 [30]
	Tanzania, Iringa					*B. globosus*					AM286288 [30]
						*B. globosus*					AM921821 [30]
	Tanzania, Ungunja, Kinyasini					*B. globosus*					AM921839 [30]
	Uganda, Lake Victoria	LV	−0.30371°N	32.28927°E	1228	*B. tropicus*	1	LV1.1	UGSB 16774	22530	MN551506
	Uganda, Lake George	LG				*B. forskalii*	2	LG1.1			HQ121586 [32]
						*B. forskalii*		LG1.2			HQ121585 [32]
	Uganda, Lake Edward	LE				*B. forskalii*	2	LE1.1			HQ121583 [32]
						*B. forskalii*		LE1.2			HQ121584 [32]
	Uganda, Lake Albert	LA				*B. forskalii*	4	LA1.1			HQ121582 [32]
						*B. forskalii*		LA1.2			HQ121580 [32]
						*B. forskalii*		LA1.3			HQ121581 [32]
						*B. forskalii*		LA1.4			HQ121587 [32]
						*B. tropicus*	2	LA1.5			HQ121564 [32]
						*B. tropicus*		LA1.6			HQ121565 [32]
						*B. tropicus*		LA1.7			GU176751 [50]
						*B. tropicus*		LA1.8			GU176750 [50]
	Uganda, Toonya, Lake Albert					*B. truncatus*					GU176749 [50]
	Uganda, Booma, Lake Albert					*B. truncatus*					GU176747 [50]
	Uganda, Katosho swamp	KS				*B. forskalii*	2	KS1.1			HQ121588 [32]
						*B. forskalii*		KS1.2			HQ121587 [32]
						*B. truncatus*					HQ121563 [32]
						*B. truncatus*					HQ121562 [32]
	Uganda, Lake Tanganyika	LT				*B. forskalii*	3	LT1.1			HQ121590 [32]
						*B. forskalii*		LT1.2			HQ121589 [32]
						*B. forskalii*		LT1.3			HQ121587 [32]
	Uganda, Albert Nile River		3.47032°N	31.92267°E		*B. globosus*					AM286291 [30]
	Uganda, Lake Kyoga		1.82235°N	33.53725°E		*B. nasutus productus*					AM921815 [30]
						*Bulinus* sp.					AM921819 [30]
	Uganda, Kahirimbi		−08205° N	30.8559 °E	1249	*Bulinus* sp.			UGSB 24296	19415	MN551577
	Uganda, Maramagambo Forest					*Bulinus* sp.					HQ121591 [32]
	Uganda, Lake Victoria		0.13707°N	33.60149°E	1135	*B. truncatus*			UGSB 16757	22513	MN551582
						*B. truncatus*			UGSB 16758	22514	MN551583
	Uganda, Nile River		1.69249°N	32.09664°E	1035	*B. truncatus*			UGSB 16760	22516	MN551585
						*B. truncatus*			UGSB 16759	22515	MN551584
	Zimbabwe, laboratory strain	ZW				*B. tropicus*	1	ZW1.1			AY282583 [37]

*Notes*: Data include the crater field region, geographical coordinates, altitude (obtained from GoogleEarth Pro 1.0.0.1), number of haplotypes, specimen code (used for the network analyses), species, DNA preparation number (prep. no.), specimen voucher (UGSB, University of Giessen Systematics and Biodiversity collection) and GenBank accession number. *Bulinus* specimens from Lake Kyaninga could not be amplified. Note that a few GenBank numbers of other authors are occurring in more than one locality because they represent same haplotypes. Locality information and geographical coordinates of GenBank sequences are provided as they were published

^a^Number of haplotypes and specimen codes are only given for specimens used in the network analyses

^b^Lakes that did not yield populations of *Bulinus*

^c^Crater lake where currently no *Bulinus* were found but sequences on GenBank were available

Since the access to the crater lakes is often made difficult by their steep escarpments, we collected snails from one to two localities per crater lake. We used scoop netting and/or dredging sampling techniques to capture snails along the edges of the access point of the lake. A maximum of 40 min sampling time per lake was used. Sampling also involved visual inspection of shoreline vegetation and hand-picking of snails. Samples were derived from depths down to a maximum of 1.5 m, covering all major habitat types present. The collected snails were fixed in 80% ethanol and stored at −20 °C for subsequent genetic analyses.

### DNA isolation, amplification and sequencing

Prior to DNA isolation, we photographed all specimens with a digital microscope system (KEYENCE VHX-2000; Keyence Deutschland GmbH, Neu-Isenburg, Germany). Genomic DNA was isolated using the CTAB method of DNA extraction [37]. In a few cases, DNA was isolated using DNeasy Blood & Tissue Kit (Qiagen, Mississauga, ON, Canada) following the provided instructions. A fragment of the mitochondrial cytochrome *c* oxidase subunit 1 (*cox*1) with a target length of 655 bp was amplified using the Folmer region primers LCO1490 [38] and COR722B [39]. PCR reactions were run according to Albrecht et al. [37]. Sanger DNA sequencing was performed on an ABI 3730xl DNA analyzer using the BigDye Terminator Kit (Life Technologies, LGC Genomics GmbH, Berlin, Germany). Vouchers (shells and DNA) are deposited in the University of Giessen Systematics and Biodiversity collection (UGSB, [40]).

### Alignment and dataset composition

The study comprised specimens from all crater lakes where *Bulinus* occurred. Furthermore, additional specimens from surrounding watershed and other major aquatic systems were included in order to better trace regional affinities. These are samples from a rice scheme and lakes Muhazi (Rwanda), Mburo, Victoria and the Nile River system in Uganda. In total, material from 43 sampling localities and 85 specimens was used for DNA analyses. Sequences were edited and aligned in BioEdit version 7.0.5 [41]. All 84 newly generated sequences were Blast-searched against the GenBank sequence database. The newly generated sequences were supplemented with all previously published relevant sequences on GenBank. The resulting dataset was aligned using the ClustalW multiple alignment tool in BioEdit after removing redundant haplotypes.

### Phylogenetic and phylogeographical analyses

Bayesian inference analysis was based on a total of 152 sequences (unique haplotypes) originating from our new sampling, as well as from GenBank data. The analysis was performed utilizing MrBayes version 3.2.2 [42] using the following settings: ngen = 4,000,000, samplefreq = 200, ‛burn-in’ = 5000 (25%) and HKY+I+Γ as the best-fit substitution model (selected using jModelTest version 2.1.4 [43] under the AIC, AICc and BIC criteria). The effective sample size (ESS) values were examined in Tracer version 1.5.0 [44] indicating for all major parameters values > 800. Statistical parsimony network analyses for all major clades found to contain crater lake specimens were conducted using TCS version 1.21 [45]. The sub-datasets were selected according to the results of the phylogenetic analysis, two specific clades were selected: Clade 1 and 2). The connection limit was set to either 95% (Clade 1) or 90% (Clade 2).

### Genetic diversity

The final cluster analysis of crater lake similarity was based on presence/absence of 31 haplotypes of *Bulinus*, the Bray-Curtis similarity measure was used (PAST version 3.22) [46].

The relationship of altitude and distribution of haplotype diversity across the crater lake fields was tested using correlation analysis implemented in PAST version 3.22 [46].

## Results

### Host species identification and diversity

Out of 58 crater lakes sampled, *Bulinus* spp. snails were found in 34 belonging to the two crater fields Bunyaruguru and Ndali-Kasenda. Although *Bulinus* spp. snails were sampled in Lake Kyaninga (Fort Portal), the samples did not yield any DNA for analysis due to technical issues. However, there is a high likelihood that the *Bulinus* spp. from this lake belong to one of the important *S. haematobium* hosts, *Bulinus globosus*, based on the shell shape (Additional file 1: Figure S1c). Lakes Rwijongo (Bunyaruguru), Mubiro and Kanyabutetere (Ndali-Kasenda) yielded *Bulinus* spp. shells only during the sampling. For Lake Rwijongo, sequences from the GenBank database were available and were included in the analyses. The rest of the crater lakes had either other gastropods or no snails at all. *Bulinus* spp. co-occurred with *Biomphalaria* spp. in 28 lakes. Three species of *Bulinus* were found, i.e. *B. forskalii*, *B. truncatus* and *B. tropicus*. The first was found in only two crater lakes (Mirambi and Kibungo), which are in close proximity located in the Ndali-Kasenda crater field. *Bulinus truncatus* exclusively occurred in Lake Kyasanduka in the Maramagambo Forest (Bunyaruguru). *Bulinus tropicus* was dominant, found in the rest of the crater lakes that harbored *Bulinus* spp. (Table 1). In addition, neither *B. forskalii* nor *B. truncatus* occurred sympatrically with *B. tropicus*. All the three *B. forskalii* and the four *B. truncatus* specimens genotyped showed the same haplotypes. *Bulinus tropicus* portrayed a high variability within and from one lake to another across the crater lakes fields. In total, this study is composed of 33 haplotypes in 34 crater lakes (one haplotype for *B. forskalii* and *B. truncatus* and 31 for *B. tropicus*).

### Phylogenetic relationships and biogeographical affinities

**Fig. 3:**
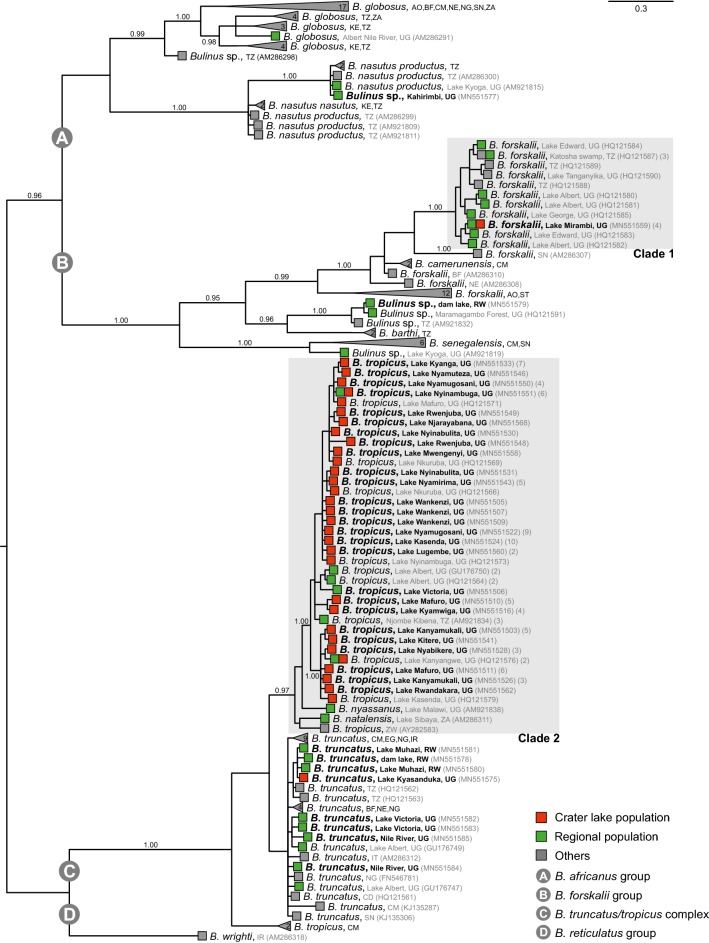
Bayesian inference phylogenetic tree for *Bulinus* spp. based on *cox*1 gene sequences. Specimens are given with locality information as to country of origin and localities in some cases. The DNA preparation numbers are provided. Crater lake names are provided and the two specific clades of *B. forskalii* (Clade 1) and *B. tropicus* (Clade 2) are highlighted with light grey boxes. Crater lake populations are represented at the end of the branch by red boxes, while regional and non-regional (= others) populations are demonstrated by green and grey boxes, respectively. The outgroup *Indoplanorbis exustus* is not shown. The tree has been partly graphically collapsed (for the full tree see Additional file 2: Figure S2). Bayesian posterior probabilities (pp) are given for deeper nodes (when pp ≥ 0.95). The scale-bar represents substitutions per site according to the model of sequence evolution. The number of individuals per haplotype is shown in parentheses for the two specific clades (for details see Figs. 4, 5)

The Bayesian inference analysis showed that *Bulinus* specimens genotyped for this study are distributed across three of the four *Bulinus* species groups/complex, specifically the *B. forskalii* and *B. africanus* groups and the *B. truncatus/B. tropicus* complex (Fig. 3). *Bulinus tropicus* from the crater lakes clustered exclusively within a highly supported *B. tropicus* clade (Clade 2, pp = 0.97, see Fig. 3 and Additional file 2: Figure S2) of the *B. tropicus/B. truncatus* complex (pp = 1.00). However, *B. tropicus* specimens from the crater lakes did not form a monophyletic group. Instead, the clade comprising the crater lake samples also included *B. tropicus* from Lake Victoria (MN551506), Lake Albert (GenBank: HQ121564, GU176750) and Njombe Kibena in Tanzania (GenBank: AM921834). Specimens from Lake Malawi (GenBank: AM921838), South Africa (GenBank: AM286311) and a laboratory strain from Zimbabwe (GenBank: AY282583) clustered in a basal position to the *B. tropicus* clade that comprised the crater lake samples. The specimen from Lake Kyasanduka and some specimens of regional populations clustered within the *B. truncatus* assemblage. These populations derived from Lake Victoria, Nile River, Lake Muhazi and other places in Rwanda, which are in close proximity to the crater field systems in western Uganda (Figs. 1, 3). The *B. truncatus* assemblage also includes populations from locations as far away as Nigeria, Cameroon, or Egypt and Burkina Faso.

The *B. forskalii* group was genetically very diverse as evidenced by the branch lengths variation with the two crater lake populations clustering with other Ugandan populations from lakes Albert, Edward and George. A distinct and highly supported clade (clade 1, pp = 1.00, see Fig. 3) also comprised populations from the Katosho swamp (Tanzania) and Lake Tanganyika (Tanzania). A *Bulinus* sp. from a dam lake connected to a rice field irrigation system in Rwanda (MN551579) belonged to the *Bulinus forskalii* group but clustered in a different subclade. It also included another *Bulinus* sp. from Maramagambo Forest, Uganda (GenBank: HQ121591), a place not far from the crater lake fields. Yet another *Bulinus* sp. from Lake Kyoga clustered with the *B. senegalensis* subclade of the *B. forskalii* group.

Another *Bulinus* sp. (MN551577) from Kahirimbi in Lake Mburo/Nakivale wetland system, about 120 km south of the crater lakes, belonged the *B. africanus* species group (Fig. 3). It was part of a clade that comprised *B. nasutus* from Lake Kyoga, Uganda and other regions in Tanzania. *Bulinus globosus* from Nile River (Moyo, Uganda) was the geographically closest occurrence of this species to the crater lakes in our dataset. Both resolution and support values were low within *B. tropicus* and *B. forskalii* clades. The phylogeographical structure for those clades were thus specifically analysed with a parsimony network analysis.

### Phylogeographical patterns

**Fig. 4:**
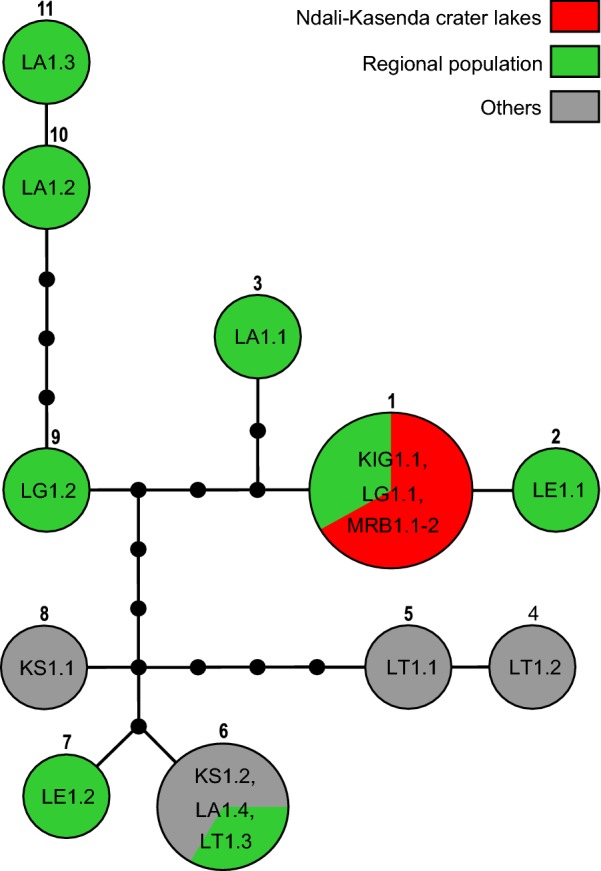
Statistical parsimony network of *cox*1 sequences for *Bulinus forskalii.* The network corresponds to the *B. forskalii* clade highlighted in Fig. 3. The connecting limit was 95%. The Ndali-Kasenda crater lake specimens are indicated in red. Haplotypes originating from regional and non-regional (= others) are in green and grey, respectively. The most probable ancestral haplotype is indicated by a bold circle

*Bulinus forskalii* from the crater lakes formed a single network with GenBank haplotypes from the surrounding lakes, i.e. Lake Albert in the north, Lake Edward in the west and Lake George in the east. A few haplotypes from regions outside Uganda, such as Lake Tanganyika and nearby Katosho swamp also appeared in the network, whereas others from Rwanda or the Maramagambo Forest east of Lake Edward did not (Fig. 4). All the three crater lake specimens represented one haplotype and together with a haplotype (GenBank: HQ121586) from Lake George formed the most probable ancestral haplotype for the network. Haplotypes from far away regions were also reconstructed as distantly related. For example, there were 11 and 10 mutational steps between haplotypes from Lake Tanganyika (GenBank: HQ121590, HQ121589). On the other hand, haplotypes from nearby regions such as Lake Edward and Lake George were reconstructed either as relatively similar (e.g. GenBank: HQ21582, HQ21583, HQ121586) or as relatively far distant in terms of mutational steps (e.g. GenBank: HQ121580).

**Fig. 5:**
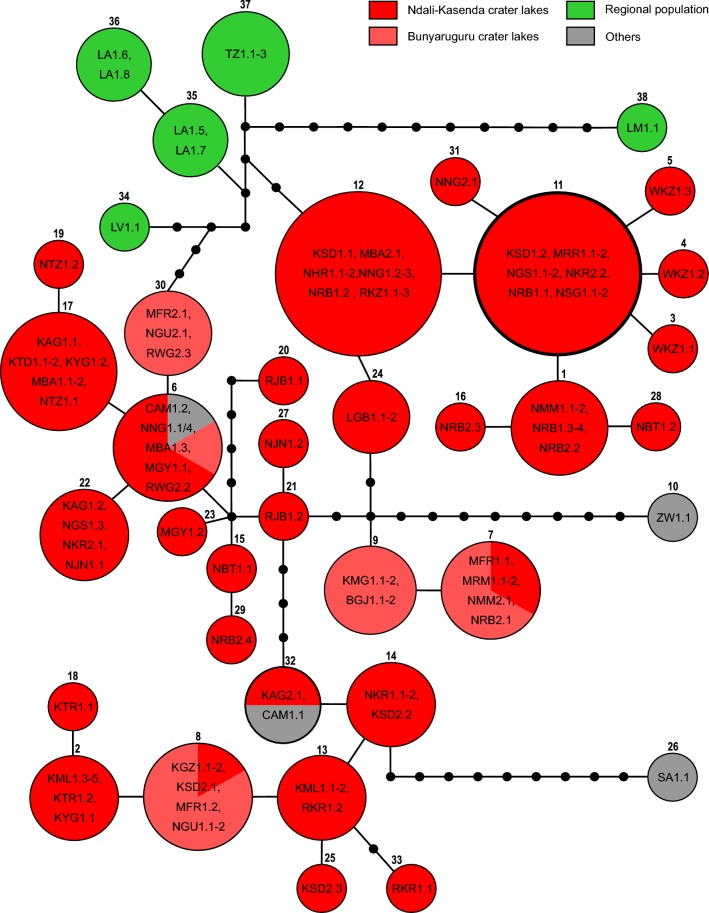
Statistical parsimony network of *cox*1 sequences for *Bulinus tropicus.* The network corresponds to the *B. tropicus* clade highlighted in Fig. 3. The connecting limit was 90%. The two crater lakes fields of Ndali-Kasenda and Bunyaruguru are coloured red and light red, respectively. Haplotypes connected but occurring in other systems are represented in green and grey for regional and non-regional (= others) populations respectively. The most probable ancestral haplotype is indicated by a bold circle

The single haplotype network of *B. tropicus* based on a 90% connection limit contained 38 haplotypes (Fig. 5). Haplotype 11 (Fig. 5) was present in six lakes in Ndali-Kasenda was reconstructed as the most probable ancestral haplotype. The haplotypes of both the Ndali-Kasenda and the Bunyaruguru crater fields were very diverse ranging from a few or no mutational steps, to as many as 17.

In most cases, haplotypes of the Ndali-Kasenda crater field were unique with a few exceptions where a haplotype was shared with either samples from the Bunyaruguru crater field (haplotypes 7 and 8 in Fig. 5), outside the region (haplotype 32 in Fig. 5) or both (haplotype 6 in Fig. 5). Lakes Kyamwiga and Bugwagyi of the Bunyaruguru crater field had exclusive haplotypes. Some lakes had quite distantly genetically related haplotypes such as MN551511, MN551510 and HQ121571, all of which are from Lake Mafuro located in the Bunyaruguru crater field. A haplotype from as far as Cameroon was identical to three crater lake samples of the Ndali-Kasenda crater field that are in close proximity to one another and Lake Rwijongo of the Bunyaruguru crater field (haplotype 6 in Fig. 5). Except for the Cameroonian, all haplotypes from outside crater lake systems are unique. These are haplotypes from Lake Victoria, Lake Albert and Tanzania. They belonged to a single group connected to crater lake haplotypes by a minimum of four mutation steps to the Ndali-Kasenda haplotypes *via* a Tanzanian haplotype (haplotype 37 in Fig. 5), Lake Albert (haplotype 35 in Fig. 5) and five mutation steps to a Bunyaruguru haplotype *via* a Lake Victoria haplotype (haplotype 34 in Fig. 5). Three haplotypes were extremely distant from the core network, i.e. samples from South Africa (haplotype 26 in Fig. 5), *B. nyassanus* from Lake Malawi (haplotype 38 in Fig. 5) and the laboratory strain from Zimbabwe (haplotype 10 in Fig. 5).

### Genetic diversity

**Fig. 6:**
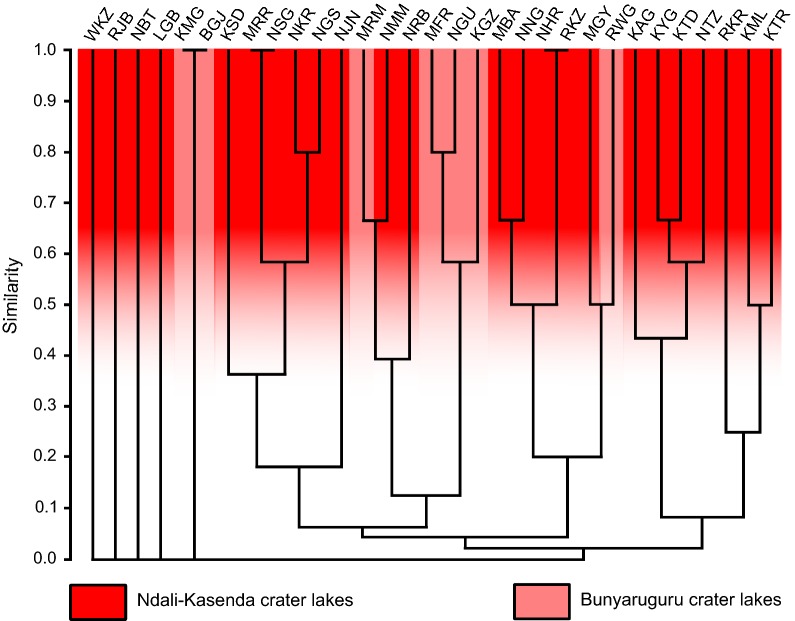
Cluster analysis of crater lake similarity based on presence/absence of 31 haplotypes of *Bulinus tropicus*. The Bray-Curtis similarity measure was used. Three letter codes refer to the respective lakes in Table 1. The haplotype matrix is given in Additional file 3: Table S1

The cluster analysis based on the presence/absence of haplotypes did not result in a clear pattern (Fig. 6). Whereas some lakes clustered together, others remained unclustered. Lakes Nyungu (NGU), Kigezi (KGZ) and Mafuro (MFR) all belong to the Bunyaruguru crater field cluster together. Other lakes belonging to that crater field such as lakes Bugwagyi (BGJ) Kyamwiga (KMG) were clustered too, whereas Rwijongo (RWG) did not cluster together with any of the two groups in the same crater field. The numerous lakes of the Ndali-Kasenda field formed three main clusters. Lakes Wankenzi (WKZ), Lugembe (LGB), Rwenjuba (RJB) and Nyinabulitwa (NBT) did not cluster to any of the other groups. Some lakes in the two crater fields tended to cluster together, for example Lake Nyamirima (NMM) with Lake Murambi (MRM) and Lake Rwijongo (RWG) with Lake Mwengenyi (MGY). Lakes that came out to be more similar according to this analysis, for example lakes Nyahira (NHR) and Rukwanzi (RKZ), or lakes Nyanswiga (NSG) and Muligamire (MRR) are not geographically related.

### Haplotype diversity across the crater lakes’ altitudinal gradient

**Fig. 7:**
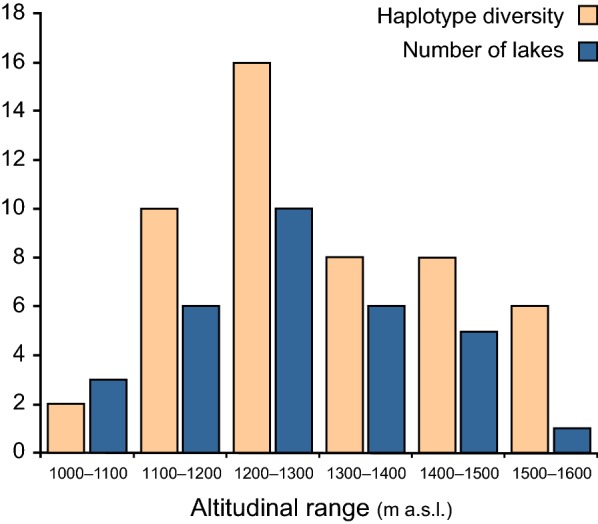
Haplotype diversity *versus* increase in altitude according to 100 m altitudinal bands for 31 unique haplotypes in 31 crater lakes

The distribution of haplotype diversity along the altitude gradient was unimodal (Fig. 7). A high haplotype diversity was realized in an altitude range between 1200 to 1300 m a.s.l., with 16 different haplotypes. Lake Nkuruba with the most haplotype diversity and situated at an altitude of 1517 m a.s.l) was represented by eight specimens and six unique haplotypes. The lakes in the Bunyaruguru crater field, which are at the lowest altitudes exhibited limited haplotype diversity (*n* = 6). Based on the present dataset, the haplotype diversity was not correlated with altitude (*r* = 0.26813, *P* = 0.12787).

## Discussion

### *Bulinus* species in the crater lakes

To date, historical records provide very restricted information on molluscs in crater lakes in Uganda [33]. Although *Bulinus tropicus* has been present there for quite a while already, not much more was known hitherto, except for the study by Nalugwa et al. [32] on *Bulinus* species complexes in the Albertine Rift, which included a few crater lakes of western Uganda. In this study, *Bulinus tropicus* by far dominates in the crater lakes, whereas *B. forskalii* was exclusively found in lakes of Mirambi and Kibungo that are in close proximity. *Bulinus forskalii* is essentially an Afrotropical species that often occurs in small and even temporary water bodies [26]. It seems less common in colder climates of highlands in eastern Africa and has been found up to 1800 m a.s.l. in Ethiopia [47]. *Bulinus forskalii* is not known to be naturally infected with *S. haematobium* (see [48]). *Bulinus tropicus* is a widespread species in eastern and southern Africa, but unlike its sibling species *B. truncatus*, is apparently not acting as intermediate host for *S*. *haematobium*, the parasite causing urogenital schistosomiasis [26, 27]. *Bulinus tropicus* is known to occur up to 2700 m a.s.l. in Kenya [26]. This species is extremely flexible ecologically, i.e. it exhibits a high tolerance towards cooling and drought conditions and even to only temporary availability of habitat. Such conditions might exist in the crater lakes of western Uganda, where extensive lake level fluctuations over seasonal periods have been documented ([49]; Tumwebaze, own observations from historical satellite images).

This ecological flexibility might be linked to another very interesting finding of the present study, which is the extremely high diversity of haplotypes of *B. tropicus* in the crater lakes. It almost matches the total range of genetic diversity hitherto known for this species [30]. Given the fact that the present study was not designed as a population study, the real variability might be even higher.

The fact that so far, the majority of the studied *Bulinus* spp. populations belonged to *B. tropicus*, does not mean *S. haematobium* strains would not occur in the entire crater lakes region. The sibling tetraploid species *B. truncatus*, a major intermediate host for *S. haematobium* in many regions of Africa, has been found in various places along the Albertine Rift [32, 50] and our dataset included populations from nearby areas in Uganda and Rwanda (Fig. 3). Although we found it exclusively in one crater lake, sympatric occurrences of the two species are possible [32]. A presence of tetraploid *B. truncatus* can therefore not be ruled out without such detailed molecular surveys such as the ones conducted in the present study. The recurrent almost complete absence from the crater lakes of *B. truncatus*, an ecologically largely flexible species with high colonizing capacity [26], remains somewhat enigmatic. This species has been found confined to altitudes of 2100 m a.s.l., rarely up to 2400 m a.s.l. in Ethiopia [26]. It was present in our study in Lake Victoria, the Nile River and Lake Muhazi, Rwanda, all locations in a radius of just *c.*250 km. *Bulinus globosus*, another potential host species, is known from the Nile River, Moyo Province, Uganda [30]. We found another *B. nasutus*-like species at the Lake Mburo-Nakivale system in southwestern Uganda. *B. nasustus* has also been found in previous work in Lake Kyoga [30] (Fig. 3). It is therefore perhaps just a matter of time and or chance until other species of *Bulinus* acting as intermediate hosts for human schistosomiasis are to be found in the crater lakes. The absence of members of the *B. africanus* group from the crater lakes is noteworthy though. Prediction as to occurrences of specific snail lineages is difficult since not only time and isolation matter, but also ecology of the small lakes. They are very different from littoral conditions in better studied large lakes such as Albert, Edward and Victoria, for which factors favoring a mitigation of the proliferation of snail populations have been determined to a much greater extent than in other lotic or lentic natural aquatic systems in East Africa [14, 51–53]. The absence of *Bulinus* in some of the studied crater lakes might be attributed to limnological conditions [18, 19]. However, 19 lakes where *Bulinus* was not collected contained other molluscs (Tumwebaze & Albrecht, unpublished data). Repeated sampling during different seasons might help constructing a more complete picture of *Bulinus* spp. communities in these lakes.

### Phylogenetic relationships and biogeographical affinities

The phylogenetic study of *Bulinus* spp. corroborated earlier findings that three major species complexes exist in the Albertine Rift region [32], although only *B. tropicus* was found to be present then in the crater lakes. Our field sampling complements the previously limited knowledge of the *Bulinus* spp. communities of the crater lakes based on the previous effort of Nalugwa et al. [32] on a smaller subset of lakes. The phylogenetic affinities of specimens genotyped from potential source populations, i.e. close geographical proximity, revealed a wide range of genetic variability which is interpreted as reflecting the high morphological and ecological flexibility of the species, as well as its extraordinary dispersal capacities. The analyses of the *B. tropicus* subclade (Fig. 3) resulted in few well-supported branches which made tracing a single origin of the crater lakes populations difficult from a phylogenetic analysis. However, the findings support the hypothesis of highly dynamic fluctuations of populations coming into the crater lakes on a potentially frequent basis. It was unexpected to find haplotypes that are known from other places far away in East Africa or even western Africa (Cameroon) in the crater lakes studied here. For the present dataset, the origin of the haplotype network was reconstructed for a haplotype occurring in six lakes of the Ndali-Kasenda lake region, likely reflecting the extraordinary diversity in the DNA data. Whereas environmental parameters might account for phenotypic plasticity observed in the *B. truncatus/B. tropicus* complex, no such direct relationships have been shown for the degree of genetic variation. It might be in fact one of the reasons why *B. tropicus* is refractory to human-infecting schistosomes [26]. Both *B. forskalii* and *B. truncatus* exhibit less genetic variation on similar geographical scales [25, 54, 55]. *Bulinus forskalii* from the crater lakes clearly belongs to a very distinct clade of Albertine Rift valley populations and colonization likely happened from nearby sources such as Lakes George, Albert and Edward. Interestingly, this clade also comprises haplotypes from further south, namely Lake Tanganyika. Other species of the *B. forskalii* group that are geographically closer, such as Lake Kyoga or Rwanda or even the Maramagambo Forest in Queen Elizabeth National Park, Uganda, do represent quite distinct lineages.

### Phylogeography and lake patterns

Since the crater lakes in western Uganda are roughly 8000 to 10,000 years-old [34], the variation observed is not likely to have developed in that setting given the general mutation rate of the molecular marker *cox*1, even if we consider potentially higher rates under tropical conditions [56]. It is worth noting that the unique haplotypes often differed by one mutational step only. We must, however, also keep in mind that the coverage of samples of *B. tropicus* throughout its vast African range is far from being representative and therefore the ‘endemicity’ of the unique haplotypes cannot be evaluated with all certainty. The variability in haplotypes might also be reflected in shell shapes as outlined by an example from Lake Mafuro, in which two distinct haplotypes corresponded to distinct shell morphs (Additional file 1: Figure S1a, b). The *Bulinus* snails from Lake Kyaninga have shells that are quite distinct from the rest in the region (Additional file 1: Figure S1c).

One question relates to colonization history, i.e. where the lineages come from. In the case of *B. tropicus*, our results identified populations from across Africa as potential sources based on genetic affinities, both from nearby source of the Victoria-Nile-Albert system or places considerably far away in Tanzania or even West Africa. The co-occurrence of distantly related haplotypes in a single lake (e.g. Nkuruba or Nyabikere) points towards multiple colonization of the same lake system likely fostered by high propagule pressure. Sharing haplotypes regionally hints towards population dispersal by passive means since most of the lakes studied have no hydrological connection. A similar pattern has been found for fishes in the Fort Portal region [57]. Machado-Schiaffino et al. [58] studying the Bunyaruguru crater lakes discovered strong genetic and morphological differentiation whereby geographically close lakes tend to be genetically more similar. Such a general trend was not obvious when comparing the lakes based on the haplotype distribution in our study.

It is important to notice that altitude reflecting climate parameters as earlier predicted [15], did not correlate with occurrence and diversity of snail populations. Rather, a more complex interplay of land use, lakes limnology, community resistance and stochasticity might account for the presence or absence of certain snail species and certain haplotypes in the crater lakes.

### Parasitological implications

This study did not find an immediate risk for urogenital schistosomiasis based on the *Bulinus* snails identified. However, the identification of up to six partly highly divergent haplotypes in small and young isolated systems such as the crater lakes in Uganda, might hint to either extremely fast evolution or multiple invasions by vectors from various source populations. This involves humans most likely. If this is the case, other species of *Bulinus* and *Biomphalaria* might also potentially be introduced. Not only is the probability of the introduction of host snails likely to increase given increased mobility of people in Uganda and international migrations, such as refugees from the crisis regions in surrounding countries, but also are such human flows capable of dispersing non-native parasite strains with them. It should be kept in mind that for example in the neighboring Nile Province of South Sudan prevalence of *S. haematobium* infection was found to be more than 70% in school children [23] and that the few modelling attempts for urogenital schistosomiasis transmission risk suggest dynamic patterns for the near future [22].

In order to establish an enhanced model of schistosomiasis prevalence in the crater lakes region, a dedicated survey of infection rates among households adjacent to the lakes that are actually using the water resources of the lakes for various purposes should be conducted. The various ways of how and to what extent water is used directly or indirectly should be assessed and quantified, as the information available with regard to these activities is very limited. The role of human (indirect) transport of both host snails and parasites is likely to be more important than previously considered, due to the important flows of human populations in the region. Movement from regions with high infection risk sites around Lake Albert and Lake Victoria or other inland water bodies infested with schistosomiasis might enhance the prevalence of schistosomiasis in the western region of Uganda. There is also need for increased surveillance of new schistosomiasis outbreaks in the crater lakes region especially at higher altitudes in the face of the projected increase in temperature in the near future [59, 60] since crater lakes have shown to be sensitive to climate change [61, 62].

A largely neglected aspect here relates to schistosomiasis as a disease in livestock. *Bulinus tropicus* and *B. forskalii* found in the crater lakes are well-known intermediate hosts for bovine schistosome species such as *S. bovis* [63–67]. This parasite is responsible for a large proportion of livestock trematode infections [68], and the parasite’s distribution overlaps largely with *S. haematobium*. Moreover, these two *Schistosoma* species have been shown to hybridize repeatedly, which triggered considerable parasitological and public health concern [69, 70]. Thus, future surveys are suggested to include molecular screening of schistosome infections [71]. *Schistosoma bovis* infections of cattle have been confirmed from western Uganda [31]. *Bulinus tropicus* and *B. forskalii* are also intermediate hosts for *Schistosoma margrebowiei* and *Calicophoron microbothrium* [72, 73], with *B. forskalii* transmitting a wide range of parasites in Ethiopia [74]. Several trematode infections have been reported in *B. forskalii* [75]. Loker et al. [76] detected cercariae of seven species from naturally infected snails in north-west Tanzania. Our study thus points to a significant concern since livelihoods of people in the crater lake region of western Uganda predominantly depend on cows, sheep and goats, which are all susceptible to schistosomiasis and other trematodiases hosted by *B. tropicus* and *B. forskalii* [77]. The crater lakes are in close proximity to nature reserves and national parks that are home to one of the most diverse primate populations in Africa. It is thus noteworthy that zoonotic schistosomiasis is a significant concern at the human-wildlife interface that is currently largely underestimated [78], which makes the crater lake region further interesting for parasitological studies in addition to the relevance for increased intestinal schistosomiasis [79].

## Conclusions

This first detailed malacological study of the crater lakes systems in western Uganda revealed a dominance of *Bulinus* species that are either not known at all (*B. tropicus* and *B. forskalii*) or not known to act as intermediate hosts of *S. haematobium*, the causative agent of human urogenital schistosomiasis in this region of Africa (*B. truncatus*). The risk of contracting this form of schistosomiasis is thus currently very low. However, potential sources for intermediate host species and known regions with high prevalence rates, have been identified in comparatively close proximities to the study region (within a radius of *c.*250 km). The epidemiology of urogenital schistosomiasis is very dynamic and there is a potential for near-future occurrence in this part of Uganda. It is thus advisable to conduct more in-depth epidemiological studies in conjunction with the activities related to intestinal schistosomiasis. There is need for coordinated effort to document the genetic diversity of schistosome intermediate hosts from small-scale (in western Uganda) to large-scale (from Uganda as a country, to east Africa and the whole of Africa), so that an effort to eradicate the parasites *via* snail control from the natural system is based on informed grounds. A cautionary note is raised in terms of the veterinary importance of the gastropod species found. They both act as intermediate host for a variety of parasites including the species causing the majority of cases of livestock schistosomiasis, *S. bovis*. The impact of this finding is potentially of major importance but currently unstudied in the region. Such studies are needed as well as investigations into factors driving the presence of hosts and parasites in regions and ecosystems so far largely neglected but with the potential of becoming major transmission sites. This is significant, especially under the projected climate changes that will shift altitudinal limits of one of the most notorious tropical diseases that continues to be a major burden especially in sub-Saharan Africa.

## Supplementary information

**Additional file 1: Figure S1.** Photographs of *Bulinus tropicus* from Lake Mafuro (a, b) and a *Bulinus* species resembling *B. globosus* of Lake Kyaninga (c) showing variation in shell morphology. Both snails from Lake Mafuro are 11 mutation steps apart in the *cox1* network.

**Additional file 2: Figure S2.** Bayesian inference phylogenetic tree for *Bulinus* spp. based on *cox1*. Specimens are given with locality information (country of origin and localities in some cases). The DNA preparation numbers are provided. Crater lake names are provided and the two specific clades of *B. forskalii* (Clade 1) and *B. tropicus* (Clade 2) are highlighted with light grey boxes. Crater lake populations are represented at the end of the branch by red boxes, while regional and non-regional (= others) populations are demonstrated by green and grey boxes, respectively. Outgroup taxa are not shown. This tree is the full version of the collapsed tree in Fig. 3. Bayesian posterior probabilities (pp) are given for deeper nodes (when pp ≥ 0.5). The scale-bar represents substitutions per site according to the applied model of sequence evolution. The number of individuals per haplotype is shown in parentheses for the two specific clades (for details see Figs. 4, 5).

**Additional file 3: Table S1.** Haplotype sequence matrix for *Bulinus tropicus* in 31 crater lakes of western Uganda. *Abbreviations*: NL, total number of haplotypes per lake; NH, total number of haplotype frequency (based on a 95% connection limit). For details of ‘lake codes’ see Table 1.

## Data Availability

Data supporting the conclusions of this article are included within the article and its additional files. The newly generated sequences were submitted to the GenBank database under the accession numbers MN551500-MN551585. The datasets generated and analysed during the present study are available in the University of Giessen Systematics and Biodiversity repository and are available upon reasonable request.

## Abbreviations

AIDS: acquired immune deficiency syndrome
a.s.l.: above sea level
*cox*1: mitochondrial cytochrome *c* oxidase subunit 1 gene
CTAB: cetyl trimethyl ammonium bromide
DNA: deoxyribonucleic acid
DRC: Democratic Republic of the Congo
MEGA: molecular evolutionary genetics analysis
NTDs: neglected tropical diseases
PAST: paleontological statistics
PCR: polymerase chain reaction
UGSB: University of Giessen Systematics and Biodiversity collection
