# Supplementary figures and images for: Molecular identification of Bulinus spp. intermediate host snails of Schistosoma spp. in crater lakes of western Uganda with implications for the transmission of the Schistosoma haematobium group parasites
Source: Parasit Vectors. 2019 Nov 27;12:565. doi: 10.1186/s13071-019-3811-2 (PMC6882369; doi:10.1186/s13071-019-3811-2)

a

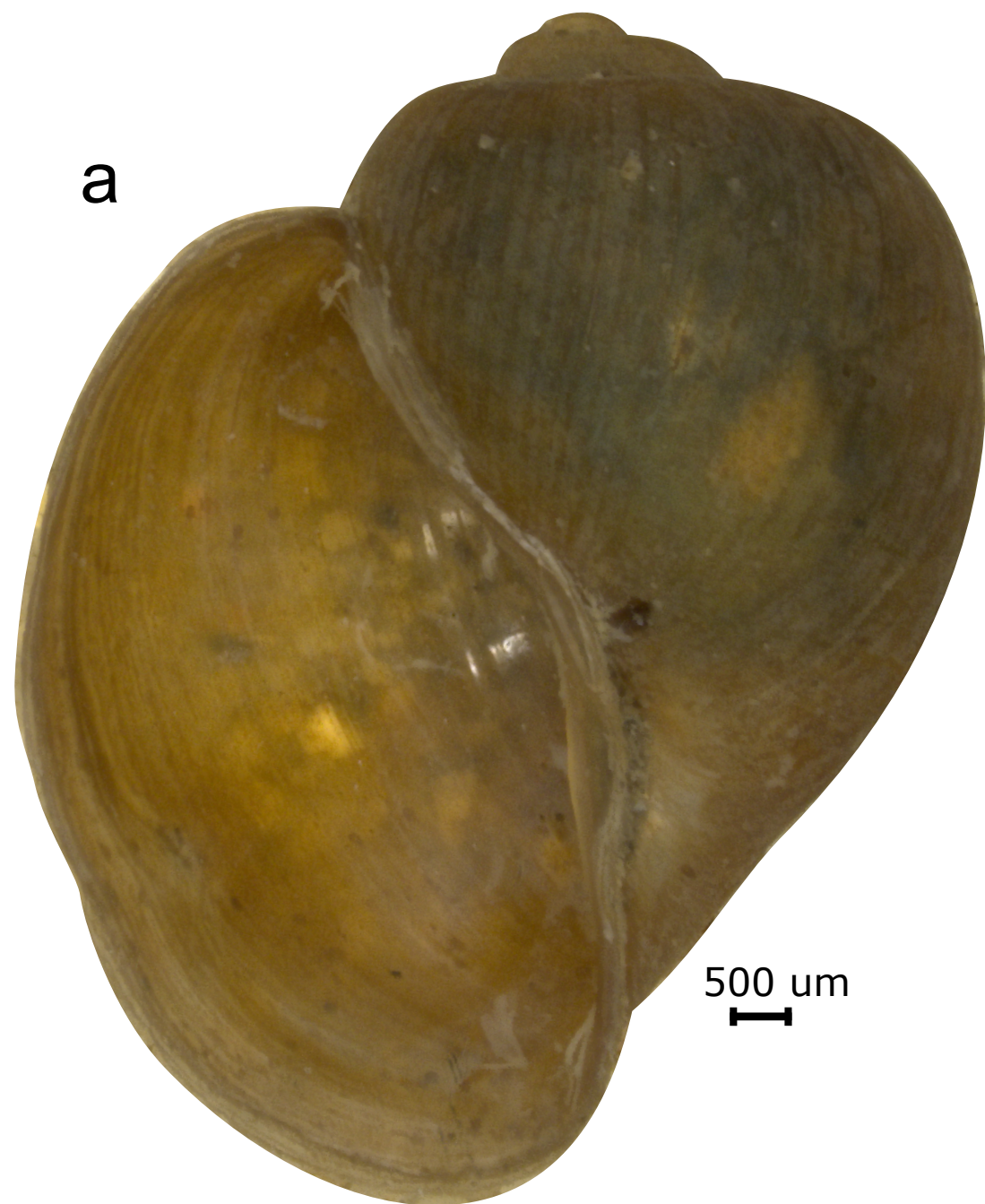

500  $\mu$ m

b

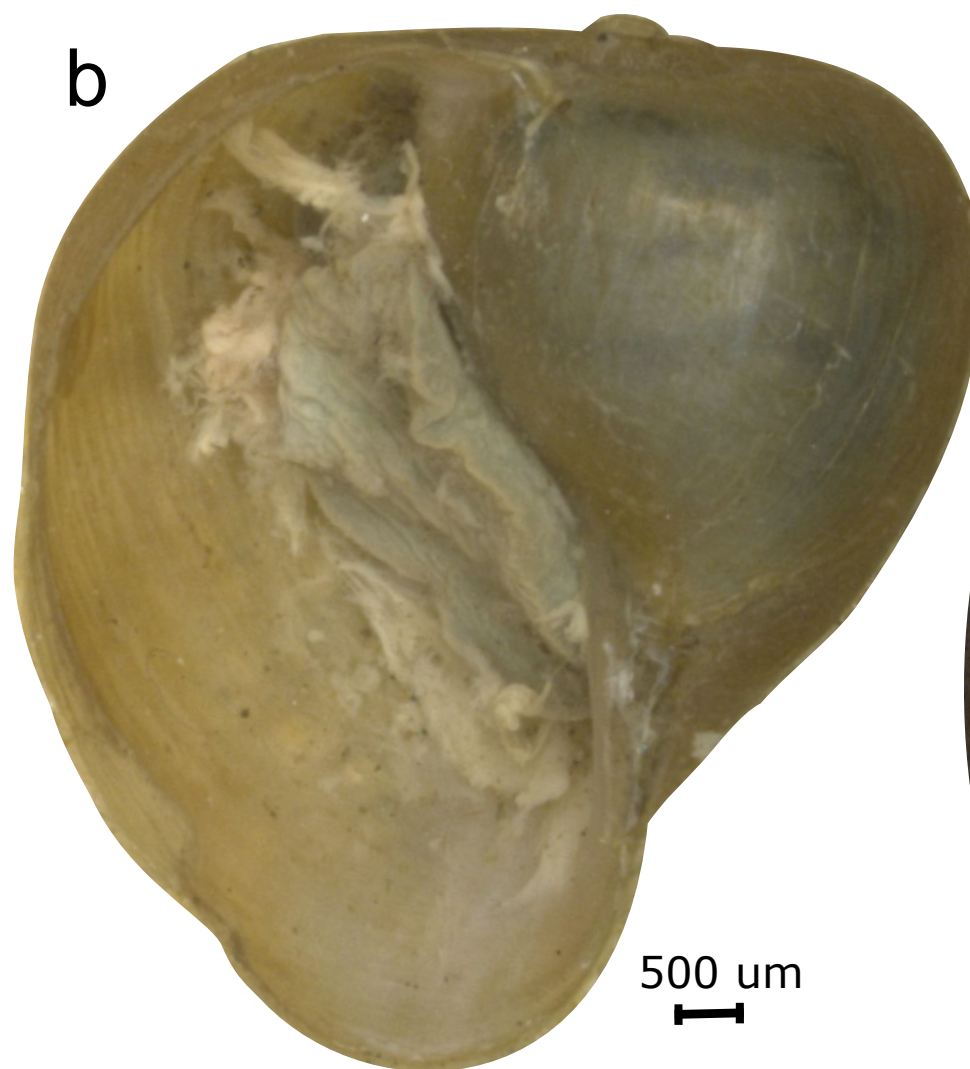

500  $\mu$ m

c

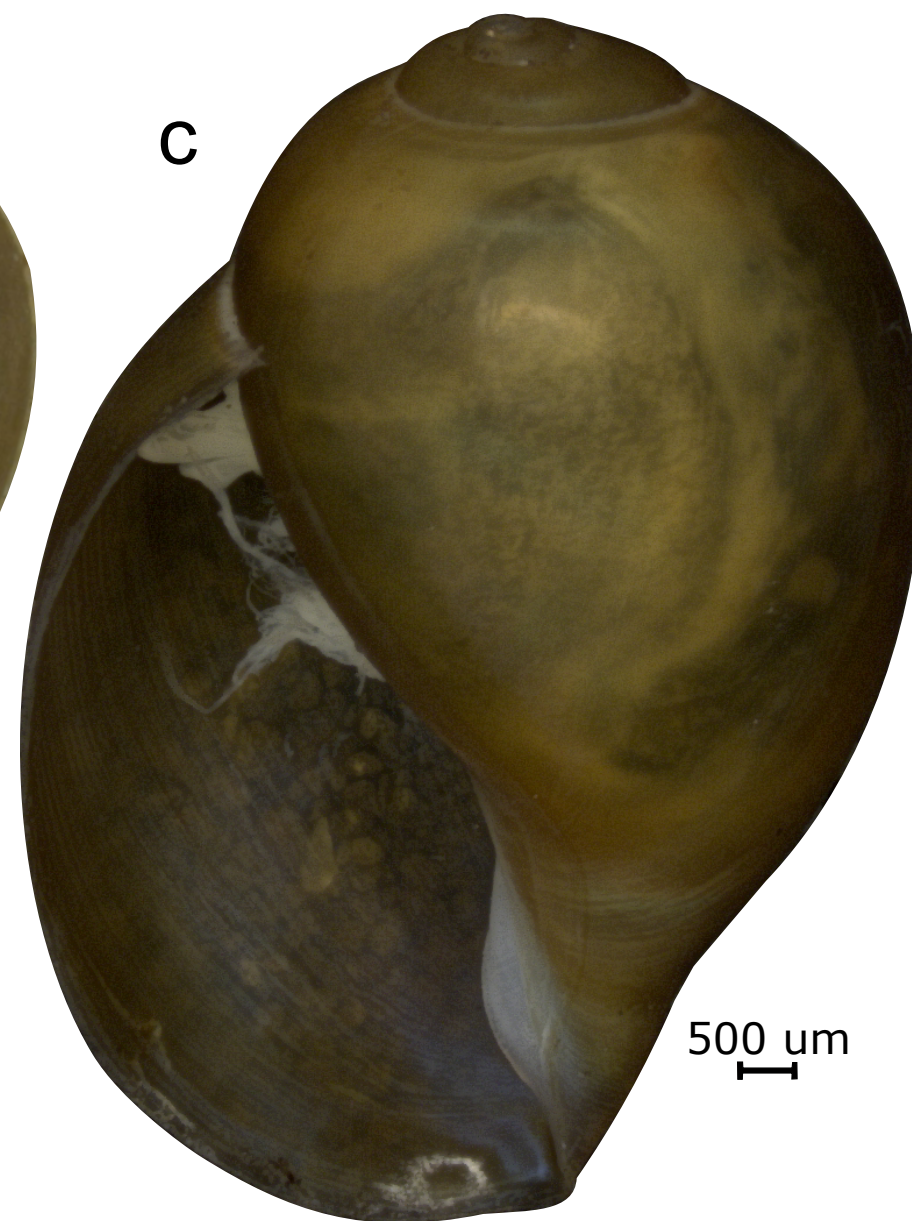

500  $\mu$ m

Supplement: Supplementary file 1 — Additional file 1: Figure S1. Photographs of Bulinus tropicus from Lake Mafuro (a, b) and a Bulinus species resembling B. globosus of Lake Kyaninga (c) showing variation in shell morphology. Both snails from Lake Mafuro are 11 mutation steps apart in the cox1 network. [file 13071_2019_3811_MOESM1_ESM.pdf]
