# Supplementary material for: Molecular identification of Bulinus spp. intermediate host snails of Schistosoma spp. in crater lakes of western Uganda with implications for the transmission of the Schistosoma haematobium group parasites
Source: Parasit Vectors. 2019 Nov 27;12:565. doi: 10.1186/s13071-019-3811-2 (PMC6882369; doi:10.1186/s13071-019-3811-2)
Supplement: Supplementary file 2 — Additional file 2: Figure S2. Bayesian inference phylogenetic tree for Bulinus spp. based on cox1. Specimens are given with locality information (country of origin and localities in some cases). The DNA preparation numbers are provided. Crater lake names are provided and the two specific clades of B. forskalii (Clade 1) and B. tropicus (Clade 2) are highlighted with light grey boxes. Crater lake populations are represented at the end of the branch by red boxes, while regional and non-regional (= others) populations are demonstrated by green and grey boxes, respectively. Outgroup taxa are not shown. This tree is the full version of the collapsed tree in Fig. 3. Bayesian posterior probabilities (pp) are given for deeper nodes (when pp ≥ 0.5). The scale-bar represents substitutions per site according to the applied model of sequence evolution. The number of individuals per haplotype is shown in parentheses for the two specific clades (for details see Figs. 4, 5). [file 13071_2019_3811_MOESM2_ESM.pdf]

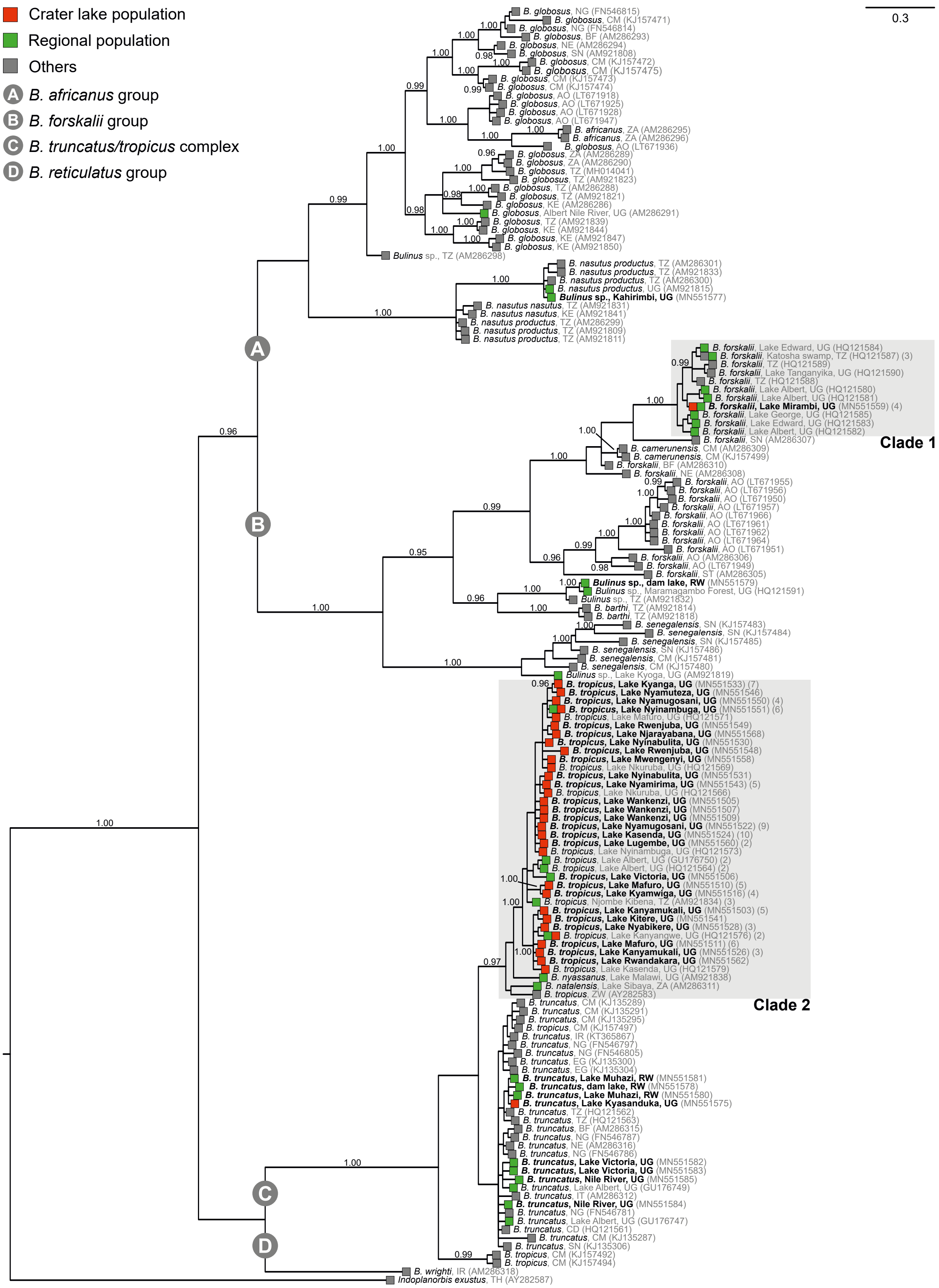
Crater lake population
Regional population
Others
A B. africanus group
B B. forskalii group
C B. truncatus/tropicus complex
D B. reticulatus group
0.3
B. globosus, NG (FN546815)
B. globosus, CM (KJ157471)
B. globosus, NG (FN546814)
B. globosus, BF (AM286293)
B. globosus, NE (AM286294)
B. globosus, SN (AM921808)
B. globosus, CM (KJ157472)
B. globosus, CM (KJ157475)
B. globosus, CM (KJ157473)
B. globosus, CM (KJ157474)
B. globosus, AO (LT671918)
B. globosus, AO (LT671925)
B. globosus, AO (LT671928)
B. globosus, AO (LT671947)
B. africanus, ZA (AM286295)
B. africanus, ZA (AM286296)
B. globosus, AO (LT671936)
B. globosus, ZA (AM286289)
B. globosus, ZA (AM286290)
B. globosus, TZ (MH014041)
B. globosus, TZ (AM921823)
B. globosus, TZ (AM286288)
B. globosus, TZ (AM921821)
B. globosus, KE (AM286286)
B. globosus, Albert Nile River, UG (AM286291)
B. globosus, TZ (AM921839)
B. globosus, KE (AM921844)
B. globosus, KE (AM921847)
B. globosus, KE (AM921850)
Bulinus sp., TZ (AM286298)
B. nasutus productus, TZ (AM286301)
B. nasutus productus, TZ (AM921833)
B. nasutus productus, TZ (AM286300)
B. nasutus productus, UG (AM921815)
Bulinus sp., Kahirimbi, UG (MN551577)
B. nasutus nasutus, TZ (AM921831)
B. nasutus nasutus, KE (AM921841)
B. nasutus productus, TZ (AM286299)
B. nasutus productus, TZ (AM921809)
B. nasutus productus, TZ (AM921811)
B. forskalii, Lake Edward, UG (HQ121584)
B. forskalii, Katosha swamp, TZ (HQ121587) (3)
B. forskalii, TZ (HQ121589)
B. forskalii, Lake Tanganyika, UG (HQ121590)
B. forskalii, TZ (HQ121588)
B. forskalii, Lake Albert, UG (HQ121580)
B. forskalii, Lake Albert, UG (HQ121581)
B. forskalii, Lake Mirambi, UG (MN551559) (4)
B. forskalii, Lake George, UG (HQ121585)
B. forskalii, Lake Edward, UG (HQ121583)
B. forskalii, Lake Albert, UG (HQ121582)
B. forskalii, SN (AM286307)
Clade 1
B. camerunensis, CM (AM286309)
B. camerunensis, CM (KJ157499)
B. forskalii, BF (AM286310)
B. forskalii, NE (AM286308)
B. forskalii, AO (LT671955)
B. forskalii, AO (LT671956)
B. forskalii, AO (LT671950)
B. forskalii, AO (LT671957)
B. forskalii, AO (LT671966)
B. forskalii, AO (LT671961)
B. forskalii, AO (LT671962)
B. forskalii, AO (LT671964)
B. forskalii, AO (LT671951)
B. forskalii, AO (AM286306)
B. forskalii, AO (LT671949)
B. forskalii, ST (AM286305)
Bulinus sp., dam lake, RW (MN551579)
Bulinus sp., Maramagambo Forest, UG (HQ121591)
Bulinus sp., TZ (AM921832)
B. barthi, TZ (AM921814)
B. barthi, TZ (AM921818)
B. senegalensis, SN (KJ157483)
B. senegalensis, SN (KJ157484)
B. senegalensis, SN (KJ157485)
B. senegalensis, SN (KJ157486)
B. senegalensis, CM (KJ157481)
B. senegalensis, CM (KJ157480)
Bulinus sp., Lake Kyoga, UG (AM921819)
B. tropicus, Lake Kyanga, UG (MN551533) (7)
B. tropicus, Lake Nyamuteza, UG (MN551546)
B. tropicus, Lake Nyamugosani, UG (MN551550) (4)
B. tropicus, Lake Nyinambuga, UG (MN551551) (6)
B. tropicus, Lake Mafuro, UG (HQ121571)
B. tropicus, Lake Rwenjuba, UG (MN551549)
B. tropicus, Lake Njarayabana, UG (MN551568)
B. tropicus, Lake Nyinabulita, UG (MN551530)
B. tropicus, Lake Rwenjuba, UG (MN551548)
B. tropicus, Lake Mwengenyi, UG (MN551558)
B. tropicus, Lake Nkuruba, UG (HQ121569)
B. tropicus, Lake Nyinabulita, UG (MN551531)
B. tropicus, Lake Nyamirima, UG (MN551543) (5)
B. tropicus, Lake Nkuruba, UG (HQ121566)
B. tropicus, Lake Wankenzi, UG (MN551505)
B. tropicus, Lake Wankenzi, UG (MN551507)
B. tropicus, Lake Wankenzi, UG (MN551509)
B. tropicus, Lake Nyamugosani, UG (MN551522) (9)
B. tropicus, Lake Kasenda, UG (MN551524) (10)
B. tropicus, Lake Lugembe, UG (MN551560) (2)
B. tropicus, Lake Nyinambuga, UG (HQ121573)
B. tropicus, Lake Albert, UG (GU176750) (2)
B. tropicus, Lake Albert, UG (HQ121564) (2)
B. tropicus, Lake Victoria, UG (MN551506)
B. tropicus, Lake Mafuro, UG (MN551510) (5)
B. tropicus, Lake Kyamwiga, UG (MN551516) (4)
B. tropicus, Njombe Kibena, TZ (AM921834) (3)
B. tropicus, Lake Kanyamukali, UG (MN551503) (5)
B. tropicus, Lake Kitere, UG (MN551541)
B. tropicus, Lake Nyabikere, UG (MN551528) (3)
B. tropicus, Lake Kanyangwe, UG (HQ121576) (2)
B. tropicus, Lake Mafuro, UG (MN551511) (6)
B. tropicus, Lake Kanyamukali, UG (MN551526) (3)
B. tropicus, Lake Rwandakara, UG (MN551562)
B. tropicus, Lake Kasenda, UG (HQ121579)
B. nyassanus, Lake Malawi, UG (AM921838)
B. natalensis, Lake Sibaya, ZA (AM286311)
B. tropicus, ZW (AY282583)
Clade 2
B. truncatus, CM (KJ135289)
B. truncatus, CM (KJ135291)
B. truncatus, CM (KJ135295)
B. tropicus, CM (KJ157497)
B. truncatus, IR (KT365867)
B. truncatus, NG (FN546797)
B. truncatus, NG (FN546805)
B. truncatus, EG (KJ135300)
B. truncatus, EG (KJ135304)
B. truncatus, Lake Muhazi, RW (MN551581)
B. truncatus, dam lake, RW (MN551578)
B. truncatus, Lake Muhazi, RW (MN551580)
B. truncatus, Lake Kyasanduka, UG (MN551575)
B. truncatus, TZ (HQ121562)
B. truncatus, TZ (HQ121563)
B. truncatus, BF (AM286315)
B. truncatus, NG (FN546787)
B. truncatus, NE (AM286316)
B. truncatus, NG (FN546786)
B. truncatus, Lake Victoria, UG (MN551582)
B. truncatus, Lake Victoria, UG (MN551583)
B. truncatus, Nile River, UG (MN551585)
B. truncatus, Lake Albert, UG (GU176749)
B. truncatus, IT (AM286312)
B. truncatus, Nile River, UG (MN551584)
B. truncatus, NG (FN546781)
B. truncatus, Lake Albert, UG (GU176747)
B. truncatus, CD (HQ121561)
B. truncatus, CM (KJ135287)
B. truncatus, SN (KJ135306)
B. tropicus, CM (KJ157492)
B. tropicus, CM (KJ157494)
B. wrighti, IR (AM286318)
Indoplanorbis exustus, TH (AY282587)
